# miR-9-5p Mediates ABCC1 to Elevate the Sensitivity of Glioma Cells to Temozolomide

**DOI:** 10.3389/fonc.2021.661653

**Published:** 2021-08-31

**Authors:** Xiang-Rui Chen, Yan-Guo Zhang, Qiang Wang

**Affiliations:** Department of Neurosurgery, The Third Affiliated Hospital of Qiqihar Medical Unversity, Qiqihar, China

**Keywords:** miR-9-5p, BCC1, glioma, temozolomide, MTT assay

## Abstract

Chemotherapy combined with surgery is an important clinical treatment for glioma, but endogenous or acquired temozolomide (TMZ) resistance can lead to poor prognosis. microRNA (miR)-9-5p acts in biological function of glioma, but the drug resistance of miR-9-5p in glioma is under exploration. The study intended to test the molecular mechanism of miR-9-5p in glioma cells. MTT assay was applied to investigate the chemosensitivity enhancement of miR-9-5p on TMZ in glioma cells U87-TMZ and U251-TMZ, and *in vivo* experiments confirmed its role on tumor growth in nude mice. The results of double luciferase reporter gene assay, qRT-PCR and WB indicated that miR-9-5p directly targeted ABCC1 (ATP binding cassette subfamily C member 1) to reduce its expressions. MTT and flow cytometry indicated that elevation of miR-9-5p or down-regulation of ABCC1 could inhibit proliferation-induced apoptosis of drug-resistant cells, and the decrease of miR-9-5p could reverse the reduction of ABCC1 on proliferation-induced apoptosis of drug-resistant cells. In vivo experiments showed that miR-9-5p could promote the anti-tumor role of TMZ. To sum up, the increase of miR-9-5p directly targets ABCC1 and may make glioma cells sensitive to TMZ.

## Introduction

Glioma is a familiar intracranial malignant tumor. It has the characteristics of rapid growth, short course of disease, easy recurrence after operation and poor prognosis (1, 2). The latest epidemiological survey of tumors shows that there are about 290,000 new malignancy patients every year, of which glioma accounts for about 40%, and recent studies have found that the onset age of glioma tends to be younger, so effective prevention and treatment programs are important for the current situation. At present, the main clinical treatments for glioma are surgery, radiotherapy and chemotherapy (3). The main chemotherapeutic drugs for glioma are cisplatin (4), carmustine (5) and teniposide (6), but the effective rate (20%) is far from ideal. In addition, the effective rate of the latest chemotherapeutic drug temozolomide (TMZ) can only reach 35% (7). And long-term radiotherapy and chemotherapy will lead to drug resistance, which is one of the main reasons for the failure of clinical treatment (8). Therefore, improving the chemosensitivity of tumor is very important to improve the survival rate of glioma patients.

microRNA (miR) is about 22nt in length (9), which can influence the transcription of downstream target genes by targeting mRNA, and play a role in inhibiting or promoting cancer (10, 11). miR can mediate target genes to regulate cell proliferation, migration and other biological processes. For example, miR-671 accelerates the proliferation of prostate cancer cells by targeting the tumor suppressor gene SOX6 (12). Other studies have found that miR-487b-5p mediates LAMP2 to regulate autophagy of lung cancer, thus increasing TMZ sensitivity (13). As the earliest discovered miR, miR-9 is expressed in many kinds of tumors, and participates in the occurrence of tumors (14). For example, miR-9 has low expression in glioma, and inhibits the proliferation of glioblastoma by regulating FOXP2. Other studies have found that miR-9 can promote the growth and metastasis of non-small cell lung cancer cells by inhibiting TGFBR2. In this research, we analyzed GSE100775 data set and found that MIR-9 was significantly decreased in glioma TMZ drug-resistant cells, suggesting that MIR-9 may play a role in glioma TMZ drug resistance. Therefore, we tested the related mechanism of miR-9 resistance in glioma TMZ, and provided a potential scheme for clinical treatment of glioma resistance.

## Methods and Materials

### GEO Chip Analysis

According to GEO database, GSE100775 chip was obtained by searching miR, glioma, TMZ keywords. We downloaded the Series Matrix File(s) file, and analyzed it with limma package. The analysis thresholds were P < 0.05 and logFC=0.5 respectively.

### Clinical Data

The tumor tissues of 27 patients with glioma from May 2015 to April 2016 were obtained, including 18 men and 9 women with an average age of 53 years. Patients have not received radiotherapy and chemotherapy after operation, and the samples were classified according to WHO classification of nervous system tumors in 2007, including 3 patients with I, 10 patients with II, 10 patients with III and 4 patients with IV. In addition, 10 brain tissues of healthy individuals hospitalized with brain trauma in our hospital were collected as the control. This research conformed to the Ethics Committee of the Third Affiliated Hospital of Qiqihar Medical Unversity and the Declaration of Helsinki, and all patients have signed informed consent (15).

### Cell Sources

U87 and U251 and normal cells HEB were obtained from ATCC and cultivated in RPMI-1640 including 10% FBS with 5% CO_2_ at 37°C. In order to construct TMZ-resistant glioma, resistant cell lines were constructed by the concentration gradient method. The concentration of TMZ was 1, 5, 10, 15 and 20μg/ml. The cell line with resistance to TMZ was called U87-TMZ/U251-TMZ. After the establishment of the cell line, it was cultured with 20μg/ml TMZ to stabilize its drug resistance for one week every month.

### Cell Transfection

miR-9-mimics, anti-miR-9, NC-mimics, anti-NC, ABCC1 (ATP binding cassette subfamily C member 1), siRNA (si-ABCC1) and si-NC were designed and synthesized by RiBoBio. Lipofectamine 2000 was applied for transfection. The lentiviral empty vector (NC-LV) expressing GFP and GFP with high miR-9 (LV-miR-9-5p) were constructed by Systems Biosciences. Virus establishment and cell transduction were carried out in basement membrane cells, and cells were obtained using puromycin (1μg/mL) and sorted using flow cytometry to maintain at 95% of GFP positive rate.

### Detection of Cell Activity

MTT assay was applied to evaluate cell viability, and the transfected cells were collected. After re-suspension, 3000 cells were inoculated in 96-well plate. After incubation for 24h, TMZ with different concentrations (20, 50, 100, 200, 300, 400, 500ml) was added. After incubation for 24h, 20μL MTT complete medium was added to each well, and then after incubation for 4h, the medium was replaced with 200μL dimethyl sulfoxide (DMSO) and mixed for 10 minutes at a wavelength of 492nm. IC 50 value of TMZ was tested using the dose response curve. The experiment was repeated three times.

### Detection of Apoptosis

Cell apoptosis was detected using annexin V-apc kit, and transfected cells were collected. FACS Calibur flow cytometer was applied for detection. U87MG cells were treated with trypsin without EDTA, rinsed with cold PBS, then resuspended in binding buffer, and dyed with annexin V-FITC and PI for 15 minutes. The numbers of annexin V-FITC positive apoptotic cells were regarded as the total number of counted cells. The experiment was repeated three times.

### qRT-PCR Detection

Total RNA was obtained, and the concentration and purity were identified. cDNA was collected using the first strand cDNA synthesis kit I (Takara Biotechnology). PCR amplification was conducted using SYBR PrimeScript™ RT-PCR kit II and ABI 7500 PCR system (ABI, USA). U6 was the internal reference of miR, and GAPDH of mRNA. The gene contents were tested by measuring Ct value and normalized by 2^- △△CT^ (16). The experiment was repeated three times.

### WB Detection

The obtained cells were lysed and the protein was tested. Equal amount of protein samples were taken using denaturing 10% SDS-PAGE and moved to PVDF. The samples were then cultivated with 5% skimmed milk powder in PBS at 4°C for 3h and with primary antibodies (Bcl-2, Bax, cle-caspase3, PARP, cyt-c, ABCC1) at 4°C overnight, and then cultivated with horseradish peroxidase (HRP) coupled secondary antibody at 37°C for 1h. Immune complexes were detected by ECL Western Blotting Kit. Image‐Pro plus software was used to analyze relative protein expression, and β -actin was applied as the internal reference. The experiment was repeated three times.

### Double Luciferase Report

The 293T cells were cultivated in a 12-well plate and then co-transfected with ABCC1-Wt or ABCC1-Mut and miR-9 mimetic or miR-9 negative control. After transfection, the cells were incubated for 24h. Cells were obtained and tested using a dual luciferase reporter gene analysis system. Luciferase activity was the ratio of firefly luciferase intensity to ranilla luciferase intensity. The experiment was repeated three times.

### Tumorigenicity of Nude Mice

Ten BALB/c athymic nude mice were from Charles River and raised without specific pathogens. In order to establish glioma xenotransplantation model, 5 U87-TMZ cells transfected with 3×10^6^ NC-LV were subcutaneously inoculated to the ventral side of nude mice, and 5 U87-TMZ cells transfected with 3×10^6^ LV-miR-9-5p were subcutaneously inoculated to the ventral side of nude mice. Four days after inoculation, five NC-LV models were randomly selected and raised with 10 mg/kg TMZ for 8 days. Fifty-six days after inoculation, all mice were executed, and tumors were resected at the same time, weighed, and photographed, and western blot was performed. The animal study was reviewed and approved by the Third Affiliated Hospital of Qiqihar Medical Unversity. The research conformed to the ethical review of animal welfare.

### Statistical Method

SPSS20.0 was applied for statistical analysis, and GraphPad 7 for visualizing the required pictures. The data were represented by Meas ± SD. Independent sample t test was applied for pair-wise comparison, one-way ANOVA for multi-group comparison, LSD-t test for afterwards comparison, repeated measurement ANOVA for multi-time point expression comparison, and Bonferroni for back testing. P< 0.05 showed a statistical difference.

## Results

### miR-9-5p Declined in Glioma

**Figure 1:**
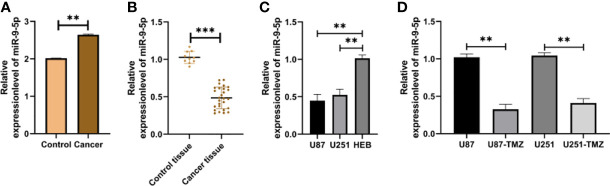
Expression of miR-9-5p in glioma. **(A)** Relative expression of miR-9-5p in TMZ-resistant cells in GEO chip. **(B)** qRT-PCR was used to detect the relative expression of miR-9-5p in tumor tissues of patients with glioma. **(C)** qRT-PCR was used to detect the relative expression of miR-9-5p in glioma cells. **(D)** The expression of miR-9-5p in TMZ-resistant glioma cells was detected by qRT-PCR. ** indicates P < 0.01; *** indicates P < 0.001.

miR-9-5p in TMZ-resistant glioma cells reduced evidently (**Figure 1A**), and miR-9-5p in glioma patients and cell lines decreased evidently (**Figures 1B, C**). In addition, by constructing TMN-resistant glioma cell lines, miR-9-5p was also declined in TMN-resistant glioma cell lines (**Figure 1D**), indicating that miR-9-5p may act in TMN-resistant glioma.

### Increase of miR-9-5p Can Hinder the Growth of TMZ-Resistant Glioma Cells

**Figure 2:**
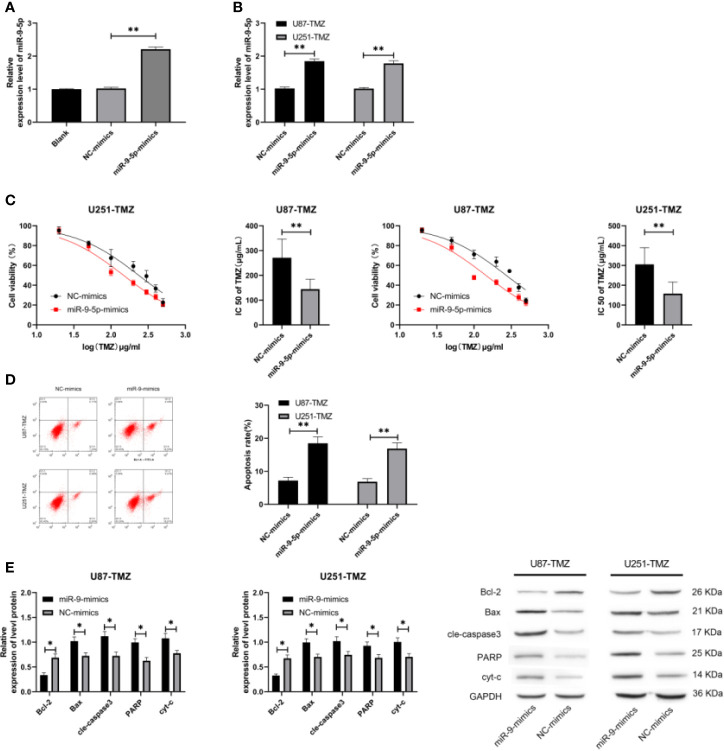
Effects of regulating miR-9-5p on biological function of TMZ-resistant glioma cells. **(A)** qRT-PCR was used to detect the relative expression of miR-9-5p after constructing miR-9-5p-mimics plasmid. **(B)** qRT-PCR was used to detect the relative expression of miR-9-5p in U87-TMZ/U251-TMZ cells transfected with miR-9-5p-mimics. **(C)** MTT assay was used to detect the changes of U87-TMZ/U251-TMZ cell activity and IC50 after transfection of miR-9-5p-mimics. **(D)** Flow cytometry was applied to detect the change of apoptosis rate of U87-TMZ/U251-TMZ cells after transfection of miR-9-5p-mimics. **(E)** WB test was used to detect the changes of Bcl-2, Bax, cle-caspase3, PARP and cyt-c protein in U87-TMZ/U251-TMZ cells after transfection of miR-9-5p-mimics. * indicates P < 0.05; ** indicates P < 0.01.

miR-9-5p-mimics plasmid (**Figure 2A**) was constructed to test miR-9-5p in TMN-resistant glioma and then transfected into U87-TMZ and U251-TMZ cells (**Figure 2B**). MTT assay was used to detect U87-TMZ and U251-TMZ after transfection of miR-9-5p-mimics. Furthermore, after further observation, the IC50 concentration also decreased evidently (**Figure 2C**), and then we found that the apoptosis rates of U87-TMZ and U251-TMZ cells transfected with miR-9-5p-mimics increased evidently (**Figure 2D**). In addition, WB experiments showed that the expression of Bax, cle-caspase3, PARP and cyt-c protein enhanced evidently after transfection of miR-9-5p-mimics, while Bcl-2 protein declined evidently (**Figure 2E**). These experiments indicated that the elevation of miR-9-5p can promote the sensitivity of glioma cells to TMZ, thus inhibiting cell growth.

### miR-9-5p Can Target ABCC1

**Figure 3:**
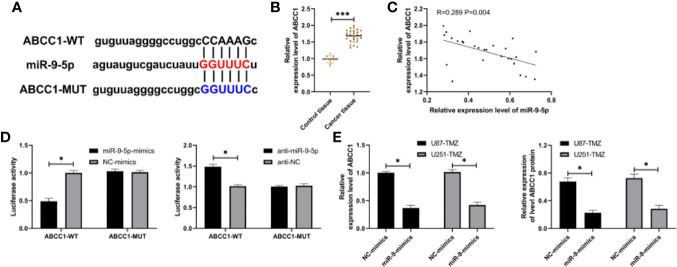
miR-9-5p has a targeted regulation relationship with ABCC1. **(A)** miR-9-5p and ABCC1 target binding site. **(B)** qRT-PCR was used to detect the relative expression of ABCC1 in tumor tissues of patients with glioma. **(C)** Correlation analysis showed that miR-9-5p was negatively correlated with ABCC1. **(D)** Double luciferase report analysis of miR-9-5p and ABCC1 targeted binding. **(E)** qRT-PCR and WB test were used to detect the relative expression of ABCC1 in U87-TMZ/U251-TMZ cells after up-regulating miR-9-5p. *indicates P < 0.05; *** indicates P < 0.001.

It has been confirmed by many reports that miR regulates downstream target genes involved in tumor drug resistance. The downstream target of miR-9-5p was established, and we found that there is a potential binding site of ABCC1 with miR-9-5p (**Figure 3A**). Therefore, ABCC1 in tumor tissues of glioma patients was also tested. qRT-PCR indicated that ABCC1 in patients increased evidently (**Figure 3B**). In addition, correlation exploration indicated that miR-9-5p had a negative correlation with ABCC1 (**Figure 3C**). miR-9-5p-mimics could hinder ABCC1-WT fluorescence activity, while anti-miR-9-5p could evidently enhance ABCC1 fluorescence activity (**Figure 3D**). qRT-PCR and WB tests indicated that ABCC1 mRNA and protein in U87-TMZ and U251-TMZ cells transfected with miR-9-mimics decreased evidently (**Figure 3E**). These experiments revealed that miR-9-5p targeted ABCC1 to act as a regulatory role.

### Decrease of miR-9-5p Affected the Sensitization of si-ABCC1 to Glioma TMZ

**Figure 4:**
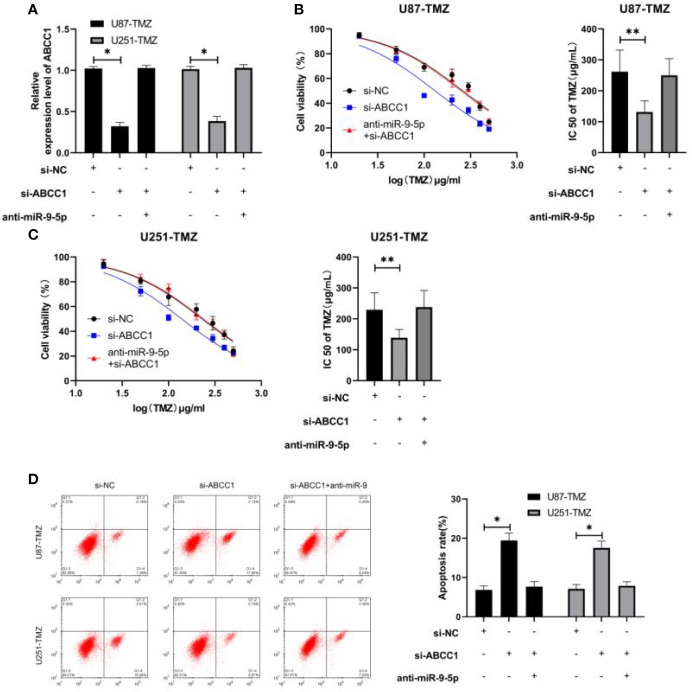
miR-9-5p can reverse the sensitization of ABCC1 to glioma TMZ. **(A)** qRT-PCR was used to detect the relative expression of ABCC1 in U87-TMZ/U251-TMZ cells after co-transfection. **(B, C)** The activity and IC50 of U87-TMZ/U251-TMZ cells were detected by MTT assay. **(D)** The change of apoptosis rate of U87-TMZ/U251-TMZ cells after co-transfection was detected by flow cytometry. * indicates P < 0.05; ** indicates P < 0.01.

We carried out a rescue experiment (**Figure 4A**) to confirm the sensitizing role of miR-9-5p/ABCC1 axis on glioma TMZ. It was found that the activity of U87-TMZ and U251-TMZ was evidently inhibited, the IC50 concentration was evidently decreased (**Figures 4B, C**), and the apoptosis was induced (**Figure 4D**), but the cell activity, IC50 concentration and apoptosis of anti- miR-9-5p and si- ABCC1 were evidently reversed, suggesting that miR-9-5p could act in the TMZ resistance mechanism of glioma by regulating ABCC1.

### Over-Expression of miR-9-5p Can Increase the Sensitivity of TMZ-Resistant Glioma

**Figure 5:**
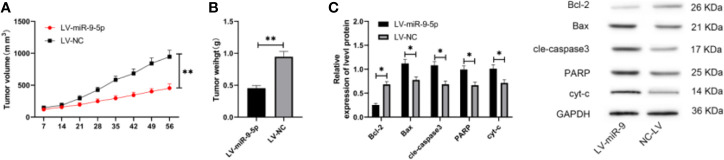
miR-9-5p can increase the sensitivity of TMZ-resistant glioma. **(A)** Tumor growth in nude mice within 56 days. **(B)** Comparison of tumor tissue quality of dead nude mice after 56 days. **(C)** Expression changes of apoptosis-related proteins in tumor tissues of nude mice. * indicates P < 0.05; ** indicates P < 0.01.

At last, we established an animal model of nude mice. Through observation, we found that the tumor volume and mass of nude mice after LV-miR-9-5p intervention were evidently lower than those of LV-NC nude mice (**Figures 5A, B**). In addition, Bax, cle-caspase3, PARP and cyt-c after LV-miR-9-5p intervention were increased, while the protein expression of Bcl-2 and ABCC1 reduced evidently (**Figure 5C**). This suggested that miR-9-5p could enhance the sensitivity of TMZ-resistant glioma to TMZ through ABCC1.

## Discussion

Glioma, as a common intracranial malignant tumor, still has defects in its treatment scheme (17). TMZ is the first choice in clinical treatment of glioma, but long-term chemotherapy will lead to drug resistance, which will eventually lead to the failure of the treatment (18). miR-9-5p decreased in glioma patients, and by restoring the content of miR-9-5p, ABCC1 could be inhibited to promote the sensitivity of TMZ-resistant glioma cells to TMZ, which may be a potential therapeutic target in clinic.

Recent studies have found that the change of miR expression plays an important role in chemical sensitivity and drug resistance mechanism (19). It has been found that miR is the key to TMZ resistance in glioma. For example, Li et al. (20) found that miR-423-5p acts in malignant phenotype and chemoresistance of glioblastoma, and another study found that miR-519a accelerates chemosensitivity and autophagy of glioblastoma by targeting STAT3/Bcl2. As the earliest discovered miR, miR-9-5p has been studied to act in many tumors (21) and nervous system diseases (22). We also analyzed GSE100775 chip and concluded that miR-9-5p in TMZ-resistant glioma cells decreased evidently. In addition, miR-9-5p in glioma tissues and cells was evidently decreased. Furthermore, miR-9-5p in drug-resistant glioma was evidently lower than that in parental cells. These experiments suggested that miR-9-5p had a correlation with TMZ resistance of glioma.

Enhancing the sensitivity of drug-resistant cells to chemotherapy drugs is one of the ways to improve the drug resistance of tumor (23, 24). In order to verify whether miR-9-5p has sensitizing effect in TMZ-resistant glioma, we established over-expression of miR-9-5p plasmids, and after transfection of miR-9-5p-mimics into drug-resistant cells, IC50 concentration of drug-resistant cells decreased evidently. We also concluded that restoring miR-9-5p in TMZ-resistant glioma cells may provide a wide range of useful methods to overcome the required drug resistance.

Each miR has multiple downstream target genes, and one gene may be adjusted by multiple miRs, thus forming a complex miR-mRNA network (25, 26). According to the prediction, we found a targeted binding of ABCC1 with miR-9-5p. Then, we detected the tumor tissues of glioma patients, and concluded that ABCC1 was enhanced in tumor tissues of patients, and correlation analysis revealed that ABCC1 had a correlation with miR-9-5p. To verify the correlation between them, ABCC1 was taken as the target of miR-9-5p. In the early studies, it was shown that ABCC1 played a sensitization role in TMZ-resistant glioma cells (27–30), revealing that miR-9-5p may adjust the resistance of glioma to TMZ through ABCC1. Then we carried out the rescue experiment. As we speculated, the IC50 of drug-resistant cells decreased after knocking down ABCC1 and the cell activity decreased, thus inducing the apoptosis. However, after co-transfection of anti-miR-9-5p and si-ABCC1, the above results were reversed. We can conclude that miR-9-5p could act in the mechanism of drug resistance of glioma cells to TMZ by regulating ABCC1.

Finally, we established a nude mouse tumor model, transfected the stable lentiviral vector LV-miR-9-5p into U87-TMZ cells, and then injected them into nude mice subcutaneously. We found that the sensitivity of nude mice to TMZ increased after LV-miR-9-5p intervention, and the tumor volume and mass decreased evidently. In addition, we also found that Bax, cle-caspase3, PARP, cyt-c and protein expression increased obviously, while Bcl-2 and ABCC1 decreased. We can confirm that increase of miR-9-5p hindered ABCC1 and increased the sensitivity of TMZ-resistant glioma cells to TMZ, thus inhibiting cell activity and inducing apoptosis.

However, this study still has some limitations. First of all, only 27 glioma samples were collected in our study, and we need to further verify whether the analysis will be biased due to the lack of data. Secondly, we did not collect TMZ-resistant glioma tumor samples in this study, and whether there are some differences between glioma tissue and drug-resistant tissue needs further detection. At last, in recent studies, it was found that long-chain non-coding RNA can regulate miR and act as a sponge for miR. We could not conclude that LncRNA regulates miR-9-5p/ABCC1 axis and then participates in glioma drug resistance. Therefore, we hope to collect more clinical samples in future studies, predict the latent binding LncRNA of miR-9-5p, and carry out experimental verification to supplement our research conclusions.

In conclusion, miR-9-5p was low in glioma, and elevation of miR-9-5p can increase the sensitivity of glioma to TMZ through ABCC1, which is a potential therapeutic target.

## Data Availability Statement

The original contributions presented in the study are included in the article/supplementary material. Further inquiries can be directed to the corresponding author.

## Ethics Statement

The studies involving human participants were reviewed and approved by Third Affiliated Hospital of Qiqihar Medical University. The patients/participants provided their written informed consent to participate in this study. The animal study was reviewed and approved by Third Affiliated Hospital of Qiqihar Medical University.

## Author Contributions

Y-GZ designed and performed experiments. X-RC designed and performed experiments, analyzed data and wrote the paper. QW designed experiments, analyzed data and wrote the paper. All authors contributed to the article and approved the submitted version.

## Conflict of Interest

The authors declare that the research was conducted in the absence of any commercial or financial relationships that could be construed as a potential conflict of interest.

## Publisher’s Note

All claims expressed in this article are solely those of the authors and do not necessarily represent those of their affiliated organizations, or those of the publisher, the editors and the reviewers. Any product that may be evaluated in this article, or claim that may be made by its manufacturer, is not guaranteed or endorsed by the publisher.
